# Supplementary material for: Impact of the COVID-19 pandemic and a supertyphoon: A quantitative study in Cebu, Philippines
Source: PLOS Glob Public Health. 2024 Dec 5;4(12):e0004008. doi: 10.1371/journal.pgph.0004008 (PMC11620371; doi:10.1371/journal.pgph.0004008)
Supplement: S1 Text — (PDF) [file pgph.0004008.s001.pdf]

STROBE Statement—Checklist of items that should be included in reports of ***cross-sectional studies***

	Item No	Recommendation	Study's characteristics
**Title and abstract**	1	(*a*) Indicate the study's design with a commonly used term in the title or the abstract	Quantitative cross sectional
		(*b*) Provide in the abstract an informative and balanced summary of what was done and what was found	Summary presented in Abstract
**Introduction**			
Background/rationale	2	Explain the scientific background and rationale for the investigation being reported	Background rationale reported
Objectives	3	State specific objectives, including any prespecified hypotheses	Specific objective reported
**Methods**			
Study design	4	Present key elements of study design early in the paper	This quantitative research was embedded in a longitudinal, prospective, population-based cohort study that evaluated the relative risk of developing virologically confirmed dengue among those who did or did not receive a single dose of CYD-TDV by previous dengue virus infections at baseline (Clinicaltrials.gov, NCT03465254) [15].
Setting	5	Describe the setting, locations, and relevant dates, including periods of recruitment, exposure, follow-up, and data collection	Setting has been reported.
Participants	6	(*a*) Give the eligibility criteria, and the sources and methods of selection of participants	The study was a sub-study of a longitudinal study, its reported.
Variables	7	Clearly define all outcomes, exposures, predictors, potential confounders, and effect modifiers. Give diagnostic criteria, if applicable	Reported.
Data sources/ measurement	8*	For each variable of interest, give sources of data and details of methods of assessment (measurement). Describe comparability of assessment methods if there is more than one group	Reported.
Bias	9	Describe any efforts to address potential sources of bias	Bias has been reported in Discussion.
Study size	10	Explain how the study size was arrived at	Reported.
Quantitative variables	11	Explain how quantitative variables were handled in the analyses. If applicable, describe which groupings were chosen and why	This is a simple descriptive analysis and has been reported.
Statistical methods	12	(*a*) Describe all statistical methods, including those used to control for confounding	Descriptive statistics.

		(*b*) Describe any methods used to examine subgroups and interactions	Reported.
		(*c*) Explain how missing data were addressed	This was a study embedded in a longitudinal study and has been reported.
		(*d*) If applicable, describe analytical methods taking account of sampling strategy	Not applicable.
		(*e*) Describe any sensitivity analyses	Not applicable.
**Results**			
Participants	13*	(a) Report numbers of individuals at each stage of study—eg numbers potentially eligible, examined for eligibility, confirmed eligible, included in the study, completing follow-up, and analysed	Reported in analysis.
		(b) Give reasons for non-participation at each stage	Part of the cohort study, selected participants all participated in the study.
		(c) Consider use of a flow diagram	Not reported.
Descriptive data	14*	(a) Give characteristics of study participants (eg demographic, clinical, social) and information on exposures and potential confounders	Reported.
		(b) Indicate number of participants with missing data for each variable of interest	Reported.
Outcome data	15*	Report numbers of outcome events or summary measures	Reported.
Main results	16	(*a*) Give unadjusted estimates and, if applicable, confounder-adjusted estimates and their precision (eg, 95% confidence interval). Make clear which confounders were adjusted for and why they were included	Simple descriptive study.
		(*b*) Report category boundaries when continuous variables were categorized	Reported.
		(*c*) If relevant, consider translating estimates of relative risk into absolute risk for a meaningful time period	Not applicable.
Other analyses	17	Report other analyses done—eg analyses of subgroups and interactions, and sensitivity analyses	Not applicable.
**Discussion**			
Key results	18	Summarise key results with reference to study objectives	Reported.
Limitations	19	Discuss limitations of the study, taking into account sources of potential bias or imprecision. Discuss both direction and magnitude of any potential bias	Reported.
Interpretation	20	Give a cautious overall interpretation of results considering objectives, limitations, multiplicity	Reported.

		of analyses, results from similar studies, and other relevant evidence	
Generalisability	21	Discuss the generalisability (external validity) of the study results	Reported.
**Other information**			
Funding	22	Give the source of funding and the role of the funders for the present study and, if applicable, for the original study on which the present article is based	Reported.

*Give information separately for exposed and unexposed groups.

Note: An Explanation and Elaboration article discusses each checklist item and gives methodological background and published examples of transparent reporting. The STROBE checklist is best used in conjunction with this article (freely available on the Web sites of PLoS Medicine at http://www.plosmedicine.org/, Annals of Internal Medicine at http://www.annals.org/, and Epidemiology at http://www.epidem.com/). Information on the STROBE Initiative is available at www.strobe-statement.org.
