# Supplementary material for: Impact of the COVID-19 pandemic and a supertyphoon: A quantitative study in Cebu, Philippines
Source: PLOS Glob Public Health. 2024 Dec 5;4(12):e0004008. doi: 10.1371/journal.pgph.0004008 (PMC11620371; doi:10.1371/journal.pgph.0004008)
Supplement: S2 Text — (PDF) [file pgph.0004008.s002.pdf]

FORM 7.1. Case report form for socio-economic and environmental profile and Impact of COVID-19 vaccination and Supertyphoon Odette (from ALL children in the cohort)

Informant Name (household head/parent or guardian)	______________________________<br>LAST NAME, FIRST NAME, MIDDLE NAME
Relationship of informant to the patient	
Patient ID	PLACE BARCODE STICKER HERE
Name of Interviewer	
Date and time of interview	__ __/__ __ __/__ __ __ __ __ __ : __ __<br>D D / M M M / Y Y Y Y H H M M

1. Socioeconomic and Environmental Profile

1.1	HOUSING and HOUSEHOLD		
1.1.1	Primary building material of the walls of the house (select one)	☐ Wood<br>☐ With screens ☐ No screens<br>☐ Cement<br>☐ With screens ☐ No screens<br>☐ Nipa<br>☐ Cloth and tarpaulin<br>☐ Others: _______	
1.1.2	Do you keep unused tires or containers with stagnant water?	☐ Yes ☐ No	
1.2	FAMILY PROFILE		
1.2.1	Number of individuals living within the household	_ _	
1.2.2	Number of children (<18 years old) within the household	_ _	
1.2.3	Individuals living in a household with household head having more than 6 years of schooling?	☐ Yes ☐ No	
1.2.4	Migrated to the current residence for the past 2 years?	☐ Yes ☐ No	
1.3	ECONOMIC PROFILE		
1.3.1	Does the family own any of the following? Tick all that apply	☐ Radio<br>☐ Television<br>☐ Refrigerator<br>☐ Bicycle<br>☐ Motorcycle	☐ Mobile phone<br>☐ Desktop or handheld computer<br>☐ Electricity<br>☐ Car<br>☐ Other (Specify.............................)
1.3.2	Estimated monthly household expenditures (in Php)	₱ ____________	

1.4	VECTOR CONTROL	
1.4.1	Does the patient use topical INSECT repellant during the day?	☐ Yes<br>If yes, tick one: ☐ Everyday<br>☐ 3-5 days/week<br>☐ Less than 3 days/week<br>☐ Other (Specify...)
		☐ No
		☐ Don't know
1.4.2	Does the household use burned mosquito coil during the day at home?	☐ Yes<br>If yes, tick one: ☐ Everyday<br>☐ 3-5 days/week<br>☐ Less than 3 days/week<br>☐ Other (Specify...)
		☐ No
		☐ Don't know
1.4.3	Does the household use insecticide spray (e.g., Baygon) during the day at home?	☐ Yes<br>If yes, tick one: ☐ Everyday<br>☐ 3-5 days/week<br>☐ Less than 3 days/week<br>☐ Other (Specify...)
		☐ No
		☐ Don't know
1.4.4	Has there been fogging in your neighborhood during the past month?	☐ Yes ☐ No ☐ Don't know/Not applicable
1.4.5	Does the household use other means to eliminate mosquitoes in the home?	☐ Yes ☐ No ☐ Don't know/Not applicable<br><br>**If yes**, specify and describe the control measure and its frequency of use<br><br>...

2. Impact of COVID-19

2.1	Questions for household head/parent or guardian	
2.1.1	Please rate the impact of COVID-19 and the lockdowns on your family (0 – mildest impact and 10 strongest impact)?	_____ Financial problems, please tick all relevant:<br>☐ Temporarily unable to work, duration _____ months<br>☐ Lost employment<br>☐ Decreased working hours / salary<br>☐ Increased expenses<br>☐ Other, please specify _____________
		_____ Mental health, please tick all relevant:<br>☐ Anxious / worried<br>☐ Depressed / sad<br>☐ Angry<br>☐ Bored<br>☐ Other, please specify _____________
		_____ Physical well-being, please tick all relevant:<br>☐ Gained weight<br>☐ Lost weight<br>☐ Had COVID-19<br>☐ Developed other illness, please specify ___________

		____ Relationships with family members, relatives, neighbors and friends, please tick all relevant:<br>☐ Decreased social contact<br>☐ Fights/disagreements<br>☐ Perceived inferiority/lack of achievement<br>☐ Other, please specify ____________
		____ COVID-19 illness or death in the family
		____ Other illness or death in the family
		____ Other problems, please specify _____________
2.1.2	Since the start of the pandemic, did anyone in your household have confirmed COVID-19?	☐ Yes, how many household members _____<br>☐ No
2.1.3	Did anyone in your household die from confirmed COVID-19?	☐ Yes, how many household members _____<br>☐ No
**2.2**	**Questions for the participant**	
2.2.1	Please rate the impact of COVID-19 and the lockdowns on you (0 – mildest impact and 10 strongest impact)?	____ Education, please tick all relevant:<br>☐ Unable to continue schooling<br>☐ Difficulty with modules or on-line work<br>☐ Miss classmates<br>☐ Other, please specify ____________
		____ Financial problems, please tick all relevant:<br>☐ No allowance or unable to work<br>☐ Increased expenses<br>☐ Other, please specify ____________
		____ Mental health, please tick all relevant:<br>☐ Anxious / worried<br>☐ Depressed / sad<br>☐ Angry<br>☐ Bored<br>☐ Other, please specify ____________
		____ Physical well-being, please tick all relevant:<br>☐ Gained weight<br>☐ Lost weight<br>☐ Had COVID-19<br>☐ Developed other illness, please specify __________
		____ Relationships with family members, relatives, neighbors and friends, please tick all relevant:<br>☐ Decreased social contact<br>☐ Fights/disagreements<br>☐ Perceived inferiority/lack of achievement<br>☐ Other, please specify ____________

2. Impact of Supertyphoon Odette

2.1	Questions for household head/parent or guardian	
2.1.1	Please rate the impact of supertyphoon Odette on your family (0 – mildest impact and 10 strongest impact)?	____ Financial problems, please tick all relevant:<br>☐ Temporarily unable to work, duration ____ months<br>☐ Lost employment<br>☐ Decreased working hours / salary<br>☐ Increased expenses<br>☐ Other, please specify ____________
		____ Mental health, please tick all relevant:<br>☐ Anxious / worried<br>☐ Depressed / sad<br>☐ Angry<br>☐ Bored<br>☐ Other, please specify ____________
		____ Living conditions:<br>☐ House damage: complete ____ or partial ____<br>☐ Had to relocate<br>☐ No electricity, duration ____ months<br>☐ Affected water supply, duration ____ months<br>☐ Other, please specify __________
