# Supplementary material for: Impact of the COVID-19 pandemic and a supertyphoon: A quantitative study in Cebu, Philippines
Source: PLOS Glob Public Health. 2024 Dec 5;4(12):e0004008. doi: 10.1371/journal.pgph.0004008 (PMC11620371; doi:10.1371/journal.pgph.0004008)
Supplement: S1 Table — (DOCX) [file pgph.0004008.s004.docx]

**Supporting Information Table 1. Demographic, socio-behavioral and economic characteristics of participants and their households**

| **Characteristics** | **N=2,630** |
| --- | --- |
|  | **Number (percentage)** |
| Age (years) |  |
| Mean (SD) | 16.4 (1.5) |
| Median (Min, Max) | 16.2 (14.2 to 20.9) |
| Sex |  |
| Female | 1,365 (51.9) |
| Male | 1,265 (48.1) |
| Place of Residence |  |
| Bogo City | 1,343 (51.1) |
| Balamban | 1,287 (48.9) |
| Housing material |  |
| Cement | 1,334 (50.7) |
| Wood | 1,296 (49.3) |
| Access to electricity |  |
| Yes | 2,591 (98.5) |
| No | 39 (1.5) |
| Number of individuals in the household |  |
| 1-4 | 668 (25.4) |
| 5-8 | 1,744 (66.3) |
| >8 | 218 (8.3) |
| Mean (SD) | 6 (2) |
| Median (Min, Max) | 5 (1, 24) |
| Number of children within household |  |
| 0-2 | 1,364 (52.1) |
| 3-5 | 1,142 (43.6) |
| >5 | 113 (4.3) |
| Mean (SD) | 3 (1.5) |
| Median (Min, Max) | 2 (0, 14) |
| Household head with > 6 years of schooling |  |
| Yes | 2,506 (95.3) |
| No | 124 (4.7) |
| Migrated to current residence in the past 2 years |  |
| Yes | 64 (2.4) |
| No | 2,566 (97.6) |
| Ownership of the following: |  |
| Radio | 1,139 (43.3) |
| Television | 1,949 (74.0) |
| Refrigerator | 1,333 (50.7) |
| Bicycle | 755 (28.7) |
| Motorcycle | 1,502 (57.1) |
| Mobile phone | 2,587 (98.4) |
| Desktop/handheld computer | 420 (16.0) |
| Car | 142 (5.4) |
| Ownership of all luxury items: |  |
| Yes | 39 (1.5) |
| No | 2,591 (98.5) |
| Ownership of at least 1 luxury item: |  |
| Yes | 2,623 (99.7) |
| No | 7 (0.3) |
| Estimated monthly household expenditure |  |
| < Php 5,000 | 56 (2.1) |
| Php 5,000-10000 | 1,603 (61.0) |
| Php 10,000-15,000 | 594 (22.6) |
| >15,000 | 377 (14.3) |
| Mean (SD) | 11,593 (6261) |
| Median (Min, Max) | 10,000 (1000 – 100,000) |
