# Supplementary material for: Impact of the COVID-19 pandemic and a supertyphoon: A quantitative study in Cebu, Philippines
Source: PLOS Glob Public Health. 2024 Dec 5;4(12):e0004008. doi: 10.1371/journal.pgph.0004008 (PMC11620371; doi:10.1371/journal.pgph.0004008)
Supplement: S2 Table — (DOCX) [file pgph.0004008.s005.docx]

**Supporting Information Table 2. Impact of COVID on the participants’ household heads or parents/guardians**

*(Scoring: 1 – mildest impact, 10 – strongest impact)*

| **Categories of impact** | **Scoring (n, %)** | **Scoring (categorical)** | **Specific impacts** |
| --- | --- | --- | --- |
| Financial problems | None: 144 (5.5)  1: 3 (0.1)  2: 13 (0.5)  3: 27 (1.0)  4: 63 (2.4)  5: 402 (15.3)  6: 275 (10.5)  7: 296 (11.2)  8: 792 (30.1)  9: 183 (7.0)  10: 432 (16.4) | None: 144 (5.5)  1 to 5: 508 (19.3)  6 to 10: 1,978 (75.2) | Increased expenses: 1,825 (69.4)  Unable to work: 1,100 (41.8),  Up to 1 month: 487 (44.2)  >1 to 3 months: 290 (26.4)  >3 to 6 months: 176 (16.0)  >6 mos to 1 year: 36 (3.3)  > 1 year: 111 (10.1)  ****Mean: 46 days, SD 106, range: 0 to 1095 days***  Decreased working hours/salary: 457 (17.4)  Lost employment: 407 (15.5) |
| Mental health | None: 44 (1.7)  1: 3 (0.1)  2: 14 (0.5)  3: 58 (2.2)  4: 91 (3.5)  5: 458 (17.4)  6: 366 (13.9)  7: 490 (18.6)  8: 477 (18.1)  9: 191 (7.3)  10: 438 (16.7) | None: 44 (1.7)  1 to 5: 624 (23.8)  6 to 10: 1,962 (74.6) | Anxious/worried: 2,223 (84.5)  Depressed/sad: 1,259 (47.9)  Bored: 1,055 (40.1)  Angry: 148 (5.6) |
| Physical well-being | None: 880 (33.5)  1: 9 (0.3)  2: 57 (2.2)  3: 174 (6.6)  4: 183 (7.0)  5: 453 (17.2)  6: 254 (9.7)  7: 252 (9.6)  8: 190 (7.2)  9: 47 (1.8)  10: 131 (5.0) | None: 880 (33.5)  1 to 5: 876 (33.3)  6 to 10: 874 (33.2) | Gained weight: 875 (33.3)  Lost weight: 847 (32.2)  Had COVID-19: 21 (0.8)  Developed other illnesses: 17 (0.7)  Hypertension: 3 (17.7)  Stroke: 3 (17.7)  Prostate disease: 2 (11.8)  Loss of appetite: 2 (11.8)  Arthritis: 1 (5.9)  Cough/colds: 1 (5.9)  Eye condition: 1 (5.9)  Fever: 1 (5.9)  Myocardial infarct: 1 (5.9)  Pneumonia: 1 (5.9)  Tuberculosis: 1 (5.9) |
| Relationships with family members, relatives, friends and neighbors | None: 498 (18.9)  1: 23 (0.9)  2: 96 (3.7)  3: 220 (8.4)  4: 192 (7.3)  5: 353 (13.4)  6: 214 (8.1)  7: 235 (8.9)  8: 264 (10.0)  9: 126 (4.8)  10: 409 (15.6) | None: 498 (18.9)  1 to 5: 884 (33.6)  6 to 19: 1,248 (47.5) | Decreased social contact: 2,024 (77.0)  Fights/disagreements: 155 (5.9)  Perceived inferiority/lack of achievement: 5 (0.2) |
| **Presence of confirmed COVID-19 within the household** | Yes: 48 (1.8)  No: 2,582 (98.2) | **Number of confirmed COVID-19 in the household** | 1: 42 (85.7)  2: 5 (10.3)  3: 1 (2.0)  5: 1 (2.0) |
| **Death from COVID-19 within the household** | Yes: 4 (0.2)  No: 2,626 (99.8) | **Number of death from COVID-19 within the household** | 1: 4 (100.0) |
