# Supplementary material for: Impact of the COVID-19 pandemic and a supertyphoon: A quantitative study in Cebu, Philippines
Source: PLOS Glob Public Health. 2024 Dec 5;4(12):e0004008. doi: 10.1371/journal.pgph.0004008 (PMC11620371; doi:10.1371/journal.pgph.0004008)
Supplement: S3 Table — (DOCX) [file pgph.0004008.s006.docx]

**Supporting Information Table 3. Impact of COVID on the adolescent participants**

*(Scoring: 1 – mildest impact, 10 – strongest impact)*

| **Categories of impact** | **Scoring (n, %)** | **Scoring (categorical)** | **Specific impacts** |
| --- | --- | --- | --- |
| Education | None: 149 (5.7)  1: 11 (0.4)  2: 49 (1.9)  3: 112 (4.3)  4: 154 (5.9)  5: 621 (23.6)  6: 350 (13.3)  7: 402 (15.3)  8: 477 (18.1)  9: 98 (3.7)  10: 207 (7.9) | None: 149 (5.7)  1 to 5: 947 (36.0)  6 to 10: 1,534 (58.3) | Difficulty with modules or online work: 2,069 (78.7)  Miss classmates: 1,133 (43.1)  Unable to continue schooling: 79 (3.0)  Work while studying: 1 (0.04) |
| Financial problems | None: 301 (11.4)  1: 16 (0.6)  2: 71 (2.7)  3: 264 (10.0)  4: 216 (8.2)  5: 614 (23.3)  6: 264 (10.0)  7: 336 (12.8)  8: 231 (8.8)  9: 81 (3.1)  10: 236 (9.0) | None: 301 (11.4)  1 to 5: 1,181 (44.9)  6 to 10: 1,148 (43.7) | Increased expenses: 1,120 (42.6)  No allowance or unable to work: 1,983 (75.4) |
| Mental health | None: 146 (5.6)  1: 9 (0.3)  2: 62 (2.4)  3: 218 (8.3)  4: 229 (8.7)  5: 550 (20.9)  6: 328 (12.5)  7: 363 (13.8)  8: 269 (10.2)  9: 138 (5.3)  10: 318 (12.1) | None: 146 (5.6)  1 to 5: 1,068 (40.6)  6 to 10: 1,416 (53.8) | Bored: 1,819 (69.2)  Anxious/worried: 1,275 (48.5)  Depressed/sad: 635 (24.1)  Angry: 148 (5.6) |
| Physical well-being | None: 801 (30.5)  1: 15 (0.6)  2: 121 (4.6)  3: 210 (8.0)  4: 238 (9.1)  5: 434 (16.5)  6: 253 (9.6)  7: 196 (7.5)  8: 174 (6.6)  9: 71 (2.7)  10: 117 (4.5) | Nome: 801 (30.5)  1 to 5: 1,018 (38.7)  6 to 10: 811 (30.8) | Gained weight: 1,120 (42.6)  Lost weight: 704 (26.8)  Had COVID-19: 21 (0.8)  Had allergy: 1 (0.04)  Anemia: 1 (0.04)  Ulcer: 1 (0.04) |
| Relationships with family members, relatives, friends and neighbors | None: 569 (21.6)  1: 21 (0.8)  2: 103 (3.9)  3: 261 (9.9)  4: 205 (7.8)  5: 370 (14.1)  6: 224 (8.5)  7: 200 (7.6)  8: 248 (9.4)  9: 115 (4.4)  10: 314 (11.9) | None: 569 (21.6)  1 to 5: 960 (36.5)  6 to 10: 1,101 (41.9) | Decreased social contact: 1,985 (75.5)  Fights/disagreements: 114 (4.3)  Perceived inferiority/lack of achievement: 4 (0.2) |
