# Supplementary material for: Impact of the COVID-19 pandemic and a supertyphoon: A quantitative study in Cebu, Philippines
Source: PLOS Glob Public Health. 2024 Dec 5;4(12):e0004008. doi: 10.1371/journal.pgph.0004008 (PMC11620371; doi:10.1371/journal.pgph.0004008)
Supplement: S4 Table — (DOCX) [file pgph.0004008.s007.docx]

**Supporting Information Table 4. Impact of Supertyphoon Rai on the on the participants’ household heads or parents/guardians**

*(Scoring: 1 – mildest impact, 10 – strongest impact)*

| **Categories of impact** | **Scoring (n, %)** | **Scoring (categorical)** | **Specific impacts** |
| --- | --- | --- | --- |
| Financial problems | None: 493 (18.8)  1: 5 (0.2)  2: 18 (0.8)  3: 46 (1.8)  4: 71 (2.7)  5: 324 (12.3)  6: 235 (8.9)  7: 333 (12.7)  8: 469 (17.8)  9: 189 (7.2)  10: 447 (17.0) | None: 493 (18.8)  1 to 5: 464 (17.6)  6 to 10: 1,673 (63.6) | Increased expenses: 1,671 (64.5)  Unable to work: 623 (23.7)  1 to 7 days: 102 (16.3)  8 to 14 days: 3 (0.5)  15 to 21 days: 300 (48.1)  22 to 30 days: 146 (23.4)  >1 to 2 months: 52 (8.3)  >2 to 3 months: 8 (1.3)  >3 months: 13 (2.1)  ****Mean: 6 days, SD 17, range: 0 to 365 days***  Decreased working hours/salary: 387 (14.7)  Lost employment: 170 (6.5)  Destruction of crops: 1 (0.04) |
| Mental health | None: 124 (4.7)  1: 3 (0.1)  2: 29 (1.1)  3: 107 (4.1)  4: 170 (6.5)  5: 517 (19.7)  6: 386 (14.7)  7: 290 (11.0)  8: 501 (19.1)  9: 122 (4.6)  10: 381 (14.5) | None: 124 (4.7)  1 to 5: 826 (31.4)  6 to 10: 1,680 (63.9) | Anxious/worried: 2,099 (79.8)  Bored: 890 (33.8)  Depressed/sad: 888 (33.8)  Angry: 144 (5.5)  Scared: 3 (0.1) |
| Living conditions | None: 47 (1.8)  1: 4 (0.2)  2: 41 (1.6)  3: 129 (4.9)  4: 152 (5.8)  5: 404 (15.4)  6: 282 (10.7)  7: 415 (15.8)  8: 495 (18.8)  9: 327 (12.4)  10: 334 (12.7) | None: 47 (1.8)  1 to 5: 730 (27.8)  6 to 10: 1,853 (70.5) | No electricity: 2,566 (97.6)  1 to 7 days: 1,236 (48.2)  8 to 14 days: 2 (0.1)  15 to 21 days: 198 (7.8)  22 to 30 days: 784 (30.6)  >1 to 2 months: 318 (12.4)  >2 to 3 months: 22 (0.9)  >3 months: 6 (0.2)  ****Mean: 20 days, SD 17, range: 0 to 120 days***  Affected water supply: 1,877 (71.4)  1 to 7 days: 738 (39.3)  8 to 14 days: 0  15 to 21 days: 477 (25.4)  22 to 30 days: 152 (8.1)  >1 to 2 months: 4 (0.2)  ****Mean: 13 days, SD 14, range: 0 to 90 days***  House damage: 1,066 (40.5)  Complete damage: 33 (3.1)  Partial damage: 1,034 (96.9)  Evacuated at the height of supertyphoon: 69 (2.6)  Had to relocate: 67 (2.6) |
