# Supplementary material for: Impact of the COVID-19 pandemic and a supertyphoon: A quantitative study in Cebu, Philippines
Source: PLOS Glob Public Health. 2024 Dec 5;4(12):e0004008. doi: 10.1371/journal.pgph.0004008 (PMC11620371; doi:10.1371/journal.pgph.0004008)
Supplement: Attachment — Submitted filename: RESPONSE TO REVIEWERS COMMENTS.docx [file pgph.0004008.s008.docx]

3^rd^ September 2024

Dear Prof. Sheikh Taslim Ali and reviewers,

Thank you very much for your suggestion and opportunity to revise our work. We have utilized your suggestions to improve our manuscript. Please see below our responses to your specific suggestions with corresponding changes in the manuscript.

We look forward to your kind consideration.

On behalf of all the authors,

Bipin Adhikari

PGPH-D-24-01211
Impact of the COVID-19 pandemic and a supertyphoon on participants of a longitudinal cohort study: A quantitative study in Cebu, Philippines
PLOS Global Public Health

Dear Dr. Adhikari,

Thank you for submitting your manuscript to PLOS Global Public Health. After careful consideration, we feel that it has merit but does not fully meet PLOS Global Public Health’s publication criteria as it currently stands. Therefore, we invite you to submit a revised version of the manuscript that addresses the points raised during the review process.

==============================

See below the reviewer comments, and additional editorial comments and suggestions for addressing the key issues on the current form of the manuscript. Realising the sufficient data availability, the study can be improved scientifically in the line of method, analyses and inferential interpretation. Refer to my suggestions in the additional editorial comments below. Also thoroughly revise the manuscript for the language and presentation with point-by-point responses to the comments from reviewers and editor for further consideration.

==============================

Please submit your revised manuscript by **21 Oct 2024**. If you will need more time than this to complete your revisions, please reply to this message or contact the journal office at [globalpubhealth@plos.org](mailto:globalpubhealth@plos.org). When you're ready to submit your revision, log on to [https://www.editorialmanager.com/pgph/](https://url.uk.m.mimecastprotect.com/s/8bOzCqVRJC88YvBtZBBWG?domain=editorialmanager.com) and select the 'Submissions Needing Revision' folder to locate your manuscript file.

Please include the following items when submitting your revised manuscript:

- A rebuttal letter that responds to each point raised by the editor and reviewer(s). You should upload this letter as a separate file labeled 'Response to Reviewers'.
- A marked-up copy of your manuscript that highlights changes made to the original version. You should upload this as a separate file labeled 'Revised Manuscript with Track Changes'.
- An unmarked version of your revised paper without tracked changes. You should upload this as a separate file labeled 'Manuscript'.

Guidelines for resubmitting your figure files are available below the reviewer comments at the end of this letter.

We look forward to receiving your revised manuscript.

Kind regards,

Sheikh Taslim Ali, M.Sc., Ph.D.
Academic Editor
PLOS Global Public Health

Journal Requirements:

1. **Please include a complete copy of PLOS’ questionnaire on inclusivity in global research in your revised manuscript. Our policy for research in this area aims to improve transparency in the reporting of research performed outside of researchers’ own country or community. The policy applies to researchers who have travelled to a different country to conduct research, research with Indigenous populations or their lands, and research on cultural artefacts. The questionnaire can also be requested at the journal’s discretion for any other submissions, even if these conditions are not met.  Please find more information on the policy and a link to download a blank copy of the questionnaire here:** [**https://journals.plos.org/globalpublichealth/s/best-practices-in-research-reporting**](https://url.uk.m.mimecastprotect.com/s/tdH9CrEZKF88QOMtzKVtt?domain=journals.plos.org)**. Please upload a completed version of your questionnaire as Supporting Information when you resubmit your manuscript.**

**AUTHORS:** Thank you for the suggestion. We have submitted the ‘questionnaire on inclusivity in global health research’

**2. Please provide your detailed Financial Disclosure statement. This is published with the article. It must therefore be completed in full sentences and contain the exact wording you wish to be published.**

****Please only choose the relevant sentences from below****

**a. Please clarify all sources of funding (financial or material support) for your study. List the grants (with grant number) or organizations (with url) that supported your study, including funding received from your institution. 
b. State the initials, alongside each funding source, of each author to receive each grant.
c. State what role the funders took in the study. If the funders had no role in your study, please state: “The funders had no role in study design, data collection and analysis, decision to publish, or preparation of the manuscript.”
d. If any authors received a salary from any of your funders, please state which authors and which funders.**

**If you did not receive any funding for this study, please simply state: “The authors received no specific funding for this work.**

**AUTHORS:** Thank you for the suggestion. We have revised/added the relevant statements related to funding in the manuscript.

**3. Figure 1: please (a) provide a direct link to the base layer of the map (i.e., the country or region border shape) and ensure this is also included in the figure legend; and (b) provide a link to the terms of use / license information for the base layer image or shapefile. We cannot publish proprietary or copyrighted maps (e.g. Google Maps, Mapquest) and the terms of use for your map base layer must be compatible with our CC-BY 4.0 license.**

**Note: if you created the map in a software program like R or ArcGIS, please locate and indicate the source of the basemap shapefile onto which data has been plotted.**

**If your map was obtained from a copyrighted source please amend the figure so that the base map used is from an openly available source. Alternatively, please provide explicit written permission from the copyright holder granting you the right to publish the material under our CC-BY 4.0 license.**

**Please note that the following CC BY licenses are compatible with PLOS license: CC BY 4.0, CC BY 2.0 and CC BY 3.0, meanwhile such licenses as CC BY-ND 3.0 and others are not compatible due to additional restrictions.**

**If you are unsure whether you can use a map or not, please do reach out and we will be able to help you. The following websites are good examples of where you can source open access or public domain maps: 
* U.S. Geological Survey (USGS) - All maps are in the public domain. (**[**http://www.usgs.gov**](https://url.uk.m.mimecastprotect.com/s/pNZdCvYqOC77wZ1UzwFjT?domain=usgs.gov)**) 
* PlaniGlobe - All maps are published under a Creative Commons license so please cite “PlaniGlobe,** [**http://www.planiglobe.com**](https://url.uk.m.mimecastprotect.com/s/_6gDCwE9PFGGln6fKBFTV?domain=planiglobe.com)**, CC BY 2.0” in the image credit after the caption. (**[**http://www.planiglobe.com/?lang=enl**](https://url.uk.m.mimecastprotect.com/s/L4chCxVqQC11Ej8HRuj-n?domain=planiglobe.com)**) 
* Natural Earth - All maps are public domain. (**[**http://www.naturalearthdata.com/about/terms-of-use/**](https://url.uk.m.mimecastprotect.com/s/6FyACy6lRHrrO3DcAC4tD?domain=naturalearthdata.com)**)**

**AUTHORS:** Thank you for the suggestion. Image was created by the authors in ESRI’s ArcGIS 10.3.1; no copyrighted material is used in this image. World Imagery ESRI Tile Layer was used as basemap located in: https://services.arcgisonline.com/ArcGIS/rest/services/World_Imagery/MapServer. Service Layer Credits: Source: Esri, Maxar, GeoEye, Earthstar Geographics, CNES/Airbus DS, USDA, USGS, AeroGRID, IGN, and the GIS User Community. Materials link: https://1drv.ms/f/s!AiOG74p-PHF0lzZqELDe80bJf8Xr?e=QOWBUZ

[CC BY 4.0](https://creativecommons.org/licenses/by/4.0/) is the appropriate license for the map.

Below are few examples of ESRI images published in PLOS ONE

[Linking management planning for coastal wetlands to potential future wave attenuation under a range of relative sea-level rise scenarios | PLOS ONE](https://journals.plos.org/plosone/article/figures?id=10.1371/journal.pone.0216695)

[The use of geographic information system and 1860s cadastral data to model agricultural suitability before heavy mechanization. A case study from Malta | PLOS ONE](https://journals.plos.org/plosone/article/figures?id=10.1371/journal.pone.0192039)

**Additional Editor Comments (if provided):

Refer to the comments from the reviewers, they differ on the current form of your manuscript. Along with these, I like you to consider following key issues of the manuscript for major revision.
- The research questions are not clearly linked with the outcome variables of their interest. Define the outcome variables and link them with the research questions in their rationale, which not clear in the text.**

**AUTHORS:** Thank you for the suggestion. We have added research question under the section ‘Data collection’ to clarify how the research question is related to the outcome variable.

**- The quantitative analyses are only descriptive in nature merely just presented the data they have collected. No inferential quantification has been made on the impact assessment to make the result more scientific. Add such inferential analyses with uncertainty in the estimates.**

**AUTHORS:** Thank you for the suggestion. Our original statistical analysis plan only intended to present the descriptive nature of the impacts by COVID-19 and typhoon Odette. Based on your suggestion, we have added the inferential analysis, in new tables (1-10). The descriptive (original) tables are now added as supplementary files.

**- As author claimed the study design is a longitudinal cohort (until Oct 2022), but no temporal assessment has been done. Authors can consider the variation in outcome variables over time across the follow up and compare against baseline scenario in 2017.**

**AUTHORS:** Thank you for the suggestion. Since this study surveyed the participants of a longitudinal cohort study, we thought to keep this information in the title and at various section, where feasible. But we agree with you, it may have added unnecessary confusion, so we have deleted the terms and have shortened it where feasible. For instance,

Revised title: Impact of the COVID-19 pandemic and a supertyphoon: A quantitative study in Cebu, Philippines

**- Not statistical analyses has been made to illustrate the individual and combined impact of two distinct disasters COVID-19 and super-typhoon specifically in the island country. Authors also could perform the risk analyses on these outcomes for these disasters and compare.**

**AUTHORS:** Thank you for the suggestion. We have added the inferential statistical analyses in the revised manuscript (tables 1-10)

**- Finally the language and presentation of the results have significant scope of improvement.**

**AUTHORS:** Thank you for the suggestion. We have revised the results based on the inferential analysis.

**Along with addressing the reviewer comments, the above listed key issues should be incorporated in the revised manuscript for further consideration. I understand it will take a bit of time, lets us know if you need more time.**

**AUTHORS:** Thank you for the suggestion and your consideration for the time.

**Reviewer #1**

**The authors assessed the impact of the COVID-19 pandemic and a supertyphoon in Cebu, Philippines. I have some comments.
1. Title: "cohort study" should NOT be emphased, since this is a cross-sectional study.**

**AUTHORS:** Thank you for the suggestion. Based on your suggestion, we have revised the title and reads as follows:

“Impact of the COVID-19 pandemic and a supertyphoon: A quantitative study in Cebu, Philippines”

**2. The results are descriptive. The authors can do some statistical inference, e.g. 1. comparing the impact between COVID-19 pandemic and the supertyphoon; 2. assessing the influential factors of COVID-19 pandemic and the supertyphoon.**

**AUTHORS:** Thank you for the suggestion. As stated above, we have added the inferential statistics based on the editor and your suggestion.

**3. Page 3: pls replace "Covid-19" with "COVID-19", deleting the "USD" before "137.7 billion".**

**AUTHORS:** Thank you for the suggestion. Revised as suggested.

**4. Section Participant: there is no need to describe vaccination.**

**AUTHORS:** Thank you for the suggestion. We have revised the section to reduce the details on vaccination.

**5. Figure 3 and Figure 4: It is better to present the percentages additionally. And what are the differences between Figure 2 and Figure 3?**

**AUTHORS:** Thank you for the question. Figure 2 represent impact of COVID-19 on household heads or parents/guardians and figure 3 represent impact of COVID-19 on the adolescent participants. We appreciate your suggestion to present the percentages additionally. Figure 3 has been revised to add the percentage.

**Figure 2**. Impact of COVID-19 on the participants’ household heads or parents/guardians

**Figure 3**. Impact of COVID-19 on the adolescent participants
**6. Table 1: pls delete "n(%)" and you can explain the meanings of figures in the footnote of the table. In addition, pls delete the colons in the first column.**

**AUTHORS:** Thank you for the suggestion. We agree the “n(%)” may have added unnecessary confusion, so we have retained “Number (percentage)”. We believe this could be self-explanatory.
**7. Tables 2-4 are not professional.**

**AUTHORS:** Thank you for the suggestion. We have added all those original tables as supplementary tables.
**8. Figure legends: pls replace "COVID" with "COVID-19".**

**AUTHORS:** Thank you for the suggestion. We have revised as suggested.

**Reviewer #2**

**I'm sorry to say that I don't think this manuscript has enough interesting findings or has advanced methodology innovation to be published.**

**AUTHORS:** Thank you for the time and evaluation. Your comments echo with other reviewers as well. We have added new tables using inferential statistics.

**Start from the title, it is very unclear what exactly the impact was of the author's primary research of interest. Reading through the full text does not help to answer this question.
Secondly, why did the authors mention COVID-19 and typhoon together in the title? I was expecting to see if the authors used some proper statistical methods to find the joint impact of COVID-19 and typhoon on something, say participant's mental health status for example. But it seems the authors just mentioned these two separately. I couldn't find anything interesting enough.
Thirdly, I don't think this manuscript presented clear causal relationship investigation on either COVID-19 or typhoon's effect. More sophisticated quantitative methods are required.**

**AUTHORS:** Thank you for your feedback. We have considered your feedback to improve our manuscript as much as possible.

**Reviewer #3**

**The study aimed to investigate the effects of COVID-19 and Supertyphoon Rai on adolescent participants and their corresponding household head or parent/guardian. The research focused on the participants' financial situation, mental health, physical well-being, and social impact. The study is well-constructed, and here are some of my comments that I hope will help the authors enhance their manuscript.**

**AUTHORS:** Thank you for your constructive evaluation. Appreciate!

**1. The topic is about the impact of COVID-19 and Supertyphoon Rai. But in the “data collection section”, it says “supertyphoon Odette” in multiple places. Please clarify or correct it.**

**AUTHORS:** Thank you for the suggestion. We have revised as suggested.

**2. Some language needs to be a little more accurate.
1) “more than two-third” means more than 66.7%. But in the below two sentences, the proportions are 63.6% and 63.9%, respectively.**

**More than two-third of the respondents (1673/2360; 63.6%)
More than two-thirds of the respondents (1680/2360; 63.9%) reported
2) “A major proportion of the respondents (1101/2630; 41.9%) had moderate to severe impact on relationship with family members, relatives, friends and neighbors. ”
“Major proportion” normally means more than 50%. When the proportion is 41.9%, suggest using “A significant/large/considerable proportion of the respondents (41.9%)...”**

**AUTHORS:** Thank you for the suggestion. We have revised it in the manuscript based on your suggestions.

**3. Figures 2, 3, and 4 are very difficult to read and interpret. Suggest using bar chart.**

**AUTHORS:** Thank you for the suggestion. We considered your suggestion and have revised the figures.
