# Supplementary material for: Impact of the COVID-19 pandemic and a supertyphoon: A quantitative study in Cebu, Philippines
Source: PLOS Glob Public Health. 2024 Dec 5;4(12):e0004008. doi: 10.1371/journal.pgph.0004008 (PMC11620371; doi:10.1371/journal.pgph.0004008)
Supplement: Attachment — Submitted filename: RESPONSE TO REVIEWERS COMMENTS.docx [file pgph.0004008.s009.docx]

28^th^ October 2024

Dear Prof. Sheikh Taslim Ali,

Thank you very much for your suggestion and opportunity to revise our work. We have utilized your suggestions to improve our manuscript. Please see below our responses to your specific suggestions with corresponding changes in the manuscript.

We look forward to your kind consideration.

On behalf of all the authors,

Bipin Adhikari

PGPH-D-24-01211R1
Impact of the COVID-19 pandemic and a supertyphoon: A quantitative study in Cebu, Philippines
PLOS Global Public Health

Dear Dr. Adhikari,

Thank you for submitting your manuscript to PLOS Global Public Health. After careful consideration, we feel that it has merit but does not fully meet PLOS Global Public Health’s publication criteria as it currently stands. Therefore, we invite you to submit a revised version of the manuscript that addresses the points raised during the review process.

Please refer to the **Additional Editor Comments** below carefully.

Please submit your revised manuscript by Nov 09 2024 11:59PM. If you will need more time than this to complete your revisions, please reply to this message or contact the journal office at [globalpubhealth@plos.org](mailto:globalpubhealth@plos.org). When you're ready to submit your revision, log on to [https://www.editorialmanager.com/pgph/](https://url.uk.m.mimecastprotect.com/s/EUaxCPMmRCK5XGQczf6CxFqPt?domain=editorialmanager.com) and select the 'Submissions Needing Revision' folder to locate your manuscript file.

Please include the following items when submitting your revised manuscript:

- A rebuttal letter that responds to each point raised by the editor and reviewer(s). You should upload this letter as a separate file labeled 'Response to Reviewers'.
- A marked-up copy of your manuscript that highlights changes made to the original version. You should upload this as a separate file labeled 'Revised Manuscript with Track Changes'.
- An unmarked version of your revised paper without tracked changes. You should upload this as a separate file labeled 'Manuscript'.

Guidelines for resubmitting your figure files are available below the reviewer comments at the end of this letter.

We look forward to receiving your revised manuscript.

Kind regards,

Sheikh Taslim Ali, M.Sc., Ph.D.
Academic Editor
PLOS Global Public Health

Journal Requirements:

**Additional Editor Comments (if provided):**

**I was expecting authors should take the comments seriously while revising their manuscripts. I found authors didn't address several key issues raised. I am still optimistic that authors could address these comments by incorporating additional simple analyses quickly before am able to have a decision on it. Along with reviewers comments, I have following comments to address.**

**AUTHORS:** Dear editor, apologies if our previous round of revision felt to be inadequate but we attempted our best to undertake the revision as diligently as possible. We are grateful for this opportunity to re-work on the revised version based on your comments below.

**1. Initial Comment: - The research questions are not clearly linked with the outcome variables of their interest. Define the outcome variables and link them with the research questions in their rationale, which not clear in the text.
AUTHORS' Respond: Thank you for the suggestion. We have added research question under the section ‘Data collection’ to clarify how the research question is related to the outcome variable.
Comment on Revised version: I suggest you can briefly utter those outcome variable with their rationale in the last paragraph of introduction for general reader first. Then for each outcome, you can link in the data collection section as you have presented now.**

**AUTHORS:** Thank you for the clarification. This may have been our oversight. We have revised the last paragraph in the introduction section to explain the outcome variable and its rationale. The revised last paragraph reads as follows:

The impacts of COVID-19 and Supertyphoon Odette are multifaceted, affecting populations in both the short and long term [14, 15]. These effects range from individual physical and mental well-being to broader social disruptions, such as employment, social cohesion, school attendance, power outages, and housing damage [16]. Exploring these impacts is critical to informing future preparedness against pandemic and disasters relevant to specific social, cultural and political context [5, 17]. Previous studies have explored the effects of COVID-19 and supertyphoon Odette on Filipinos, but they were limited by their narrow focus and small sample sizes [18-23]. The main objective of this study was to explore the multifaceted impact of COVID-19 and supertyphoon Odette on Filipino adolescents and their corresponding household head, parent, or guardian.

**2. Initial Comment:- The quantitative analyses are only descriptive in nature merely just presented the data they have collected. No inferential quantification has been made on the impact assessment to make the result more scientific. Add such inferential analyses with uncertainty in the estimates.
AUTHORS' Respond: Thank you for the suggestion. Our original statistical analysis plan only intended to present the descriptive nature of the impacts by COVID-19 and typhoon Odette. Based on your suggestion, we have added the inferential analysis, in new tables (1-10). The descriptive (original) tables are now added as supplementary files.
Comment on Revised version:
- First, do you think all 10 tables (table 1 to 10) should be the main text figure? I suggest select maximum 2-4 key table for main text and rest should be in supplementary or try to combine these tables in case. I am confused with the table numbering only two tables (table S1 and S2) are cited in the main text but they listed 4 tables as supporting information (page 15) with typo in their labelling. Make sure all tables and figures should be discusses and cited in the main text.**
**AUTHORS:** Thank you for the suggestion. We have combined the tables 1-10 based on the respondents’ type with corresponding domains within the tables as follows:

**Table 1. Impacts of COVID-19 to the household**

Domain-I Financial impacts

Domain-II: Physical wellbeing impacts

Domain-III: Relational impacts

**Table 2. Impacts of COVID-19 to the cohort participant**

Domain-I Schooling impacts

Domain-II: Financial impacts

Domain-III: Mental health impacts

Domain-IV: Physical wellbeing impacts

Domain-V: Relational impacts

**Table 3. Impacts of Supertyphoon Odette to the household**

Domain-I Financial impacts

Domain-II: Mental health impacts

**- Secondly, including p-value in the table looks better. I understand authors performed several tests including Chi-squared tests, Fisher’s exact and the Mann−Whitney U test based on the data. I suggest to provide the details of the test used with rationale in foot notes of each table.**

**AUTHORS:** Thank you for the suggestion. We have added symbols for each p-value with footnotes in each of the table explaining the reasons for these specific tests that reads as follows:

*Chi-squared test for categorical variables with cell sizes more than 50; **Fisher Exact test for categorical variables with cell sizes less than 50; ^¶^Mann Whitney U test for median values between two categories.

**3. Initial Comment:- As author claimed the study design is a longitudinal cohort (until Oct 2022), but no temporal assessment has been done. Authors can consider the variation in outcome variables over time across the follow up and compare against baseline scenario in 2017.
AUTHORS' Respond: Thank you for the suggestion. Since this study surveyed the participants of a longitudinal cohort study, we thought to keep this information in the title and at various section, where feasible. But we agree with you, it may have added unnecessary confusion, so we have deleted the terms and have shortened it where feasible. For instance, Revised title: Impact of the COVID-19 pandemic and a supertyphoon: A quantitative study in Cebu, Philippines.
Comment on Revised version: It is not only the question of title, my suggestion to see the outcome status temporarily (under each follow ups) where the baseline scenario can be of 2017. As the study was conducted longitudinally, why not considering the temporal analyses on the outcomes using simply the consecutive followup data. I am not convinced with the author response and it is not cross-sectional study.
AUTHORS:** Thank you for the comment. Apologies for the confusion. This sub-study is only a part of the longitudinal study but was a cross-sectional survey. We have revised under ‘study context’ to explicitly report it as a ‘cross-sectional survey’.

**4. Initial Comment:- No statistical analyses has been made to illustrate the individual and combined impact of two distinct disasters COVID-19 and super-typhoon specifically in the island country. Authors also could perform the risk analyses on these outcomes for these disasters and compare.
AUTHORS' Respond: Thank you for the suggestion. We have added the inferential statistical analyses in the revised manuscript (tables 1-10)
Comment on Revised version: Here I meant to quantify the risk. Tables 1-10 could help to identify the risk factors, but any way to quantify them, which could be helpful in the line of interventions.**

**AUTHORS:** Thank you for the suggestion. With due diligence to your suggestions, our study collected data among three discrete respondent types/levels (1. Impacts of COVID-19 to the household; 2. Impacts of COVID-19 to the cohort participants; and 3. Impacts of Supertyphoon Odette to the households). Because of the heterogeneity of the respondent types, we could not merge all data to compare with outcome variables. Secondly, since there were multiple outcomes we were interested in, we considered to explore the impacts in terms of these outcomes rather than combining/reducing these outcomes into a single dependent variable. Thirdly, there were differences in the domains within each type of respondents which we believed would best fit as independent tables. Lastly, because of the nature of these discrete datasets (domains and respondent types), their impacts may benefit when presented as descriptive and by large, through within respondents’ comparative analysis. We hope we are able to explain our rationale for our limitations.

**5. Fig 1 is missing in revised manuscript, is it by mistake?**

**AUTHORS:** Thank you for the question. It was an oversight. We have added the figure 1 in revised submission.

**6. The Fig 2-4, revise the x-lable as "Number of participants (%)" and can be combined in one figure for better integrated comprehension.**

**AUTHORS:** Thank you for the suggestion. We have revised the figures as you suggested.

**7. Finally, the text should be revised for English and presentation (for general reader).**

**AUTHORS:** Thank you for the suggestion. We have our manuscript proof-read and edited by a native English speaker.
